# Severe cardiomyopathy due to permanent junctional reciprocating tachycardia: recovery after catheter ablation?—a case report

**DOI:** 10.1093/ehjcr/ytag089

**Published:** 2026-02-03

**Authors:** Shaojie Chen, Ardan Muammer Saguner, Firat Duru, Boris Schmidt, Julian K R Chun

**Affiliations:** Rhythmology and Clinical Cardiac Electrophysiology, Klinik Internal Medicine B (Cardiology, Angiology, Pneumology and Internal Intensive Care Medicine), German Centre for Cardiovascular Research (DZHK), University Medicine Greifswald, Ferdinand-Sauerbruch-Straße, Greifswald 17475, Germany; Department of Cardiology, University Heart Center, University Hospital Zurich, Rämistrasse 100, 8091 Zurich, Switzerland; Center for Translational and Experimental Cardiology (CTEC), University of Zurich, Rämistrasse 71, 8006 Zurich, Switzerland; Department of Cardiology, University Heart Center, University Hospital Zurich, Rämistrasse 100, 8091 Zurich, Switzerland; Center for Translational and Experimental Cardiology (CTEC), University of Zurich, Rämistrasse 71, 8006 Zurich, Switzerland; Cardioangiologisches Centrum Bethanien (CCB), Kardiologie, Medizinische Klinik III, Agaplesion Markus Krankenhaus, Akademisches Lehrkrankenhaus der Goethe-Universität, Wilhelm-Epstein-Straße 4, 60431 Frankfurt am Main, Germany; Cardioangiologisches Centrum Bethanien (CCB), Kardiologie, Medizinische Klinik III, Agaplesion Markus Krankenhaus, Akademisches Lehrkrankenhaus der Goethe-Universität, Wilhelm-Epstein-Straße 4, 60431 Frankfurt am Main, Germany; Die Sektion Medizin, Universität zu Lübeck, Ratzeburger Allee 160, 23562 Lübeck, Germany

**Keywords:** Catheter ablation, Permanent junctional reciprocating tachycardia, Tachycardia-induced severe cardiomyopathy, Reversal of left ventricular dysfunction, Case report

## Abstract

**Background:**

Permanent junctional reciprocating tachycardia (PJRT) is a rare form of orthodromic atrioventricular reciprocating tachycardia involving a slowly conducting, decremental concealed accessory pathway. Its incessant nature may result in tachycardia-induced cardiomyopathy, which is reversible with timely rhythm control.

**Case summary:**

A 41-year-old patient presented with progressive exertional dyspnoea and reduced exercise capacity. Continuous electrocardiogram monitoring revealed an incessant long RP narrow QRS tachycardia (heart rate 135 b.p.m.). Echocardiography demonstrated severe LV systolic dysfunction (LVEF 14%) and LV dilatation (LVEDD 63 mm). Coronary angiography and cardiac MRI excluded relevant structural or infiltrative cardiomyopathy. Electrophysiological study confirmed PJRT using a posteroseptal concealed accessory pathway. Catheter ablation at the site of earliest atrial activation resulted in immediate termination of tachycardia and loss of accessory pathway conduction. Post-ablation echocardiography showed early improvement in LVEF to 26%. The patient was discharged on guideline-directed medical therapy for heart failure. At 6-month follow-up, he remained asymptomatic, with no arrhythmia recurrence and recovery of LVEF to 50% with normalization of LV dimensions.

**Discussion:**

This case highlights the reversibility of severe cardiomyopathy secondary to incessant PJRT. Even in cases with severe LV dysfunction, timely identification and ablation of the accessory pathway can achieve recovery of LV function. Careful electrophysiologic evaluation is essential to differentiate PJRT from other long RP tachycardias and guide curative therapy.

Learning points

- Permanent junctional reciprocating tachycardia is a rare cause of incessant long RP narrow QRS tachycardia that may lead to *severe* tachycardia-induced cardiomyopathy if not promptly recognized.
- Catheter ablation of the concealed accessory pathway is the treatment of choice, offering a high likelihood of arrhythmia cure and reversal of ventricular dysfunction.
- Early electrophysiologic evaluation should be considered in unexplained LV dysfunction with persistent supraventricular tachycardia, as timely intervention can restore cardiac function.

## Introduction

Permanent junctional reciprocating tachycardia (PJRT) is a rare form of orthodromic atrioventricular re-entrant tachycardia (AVRT), characterized by an incessant, long RP tachycardia utilizing a slowly conducting, concealed accessory pathway, typically with decremental retrograde conduction. Unlike conventional orthodromic AVRT involving a fast-conducting accessory pathway, PJRT often presents with relatively slow, persistent tachycardia rates, leading to delayed recognition.^1^

If unrecognized or left untreated, the incessant tachycardia and chronic tachycardia burden can result in tachycardia-induced cardiomyopathy (TIC), manifesting as left ventricular (LV) systolic dysfunction and heart failure. This form of cardiomyopathy is potentially reversible with appropriate treatment, underscoring the importance of early identification.^2,3^

While pharmacologic therapy (such as beta-blockers or antiarrhythmics) may transiently control the heart rate, it is often insufficient to achieve long-term suppression. Catheter ablation of the accessory pathway is considered the treatment of choice, offering a high likelihood of definitive cure and reversal of LV dysfunction.^1^

We present a case of PJRT leading to severely reduced LV ejection fraction, successfully treated with catheter ablation, resulting in recovery of cardiac function. This case highlights the importance of recognizing PJRT as an uncommon but reversible cause of severely dilated cardiomyopathy and reinforces the role of early curative therapy to prevent or reverse severe heart failure.

## Summary figure

**Figure:**
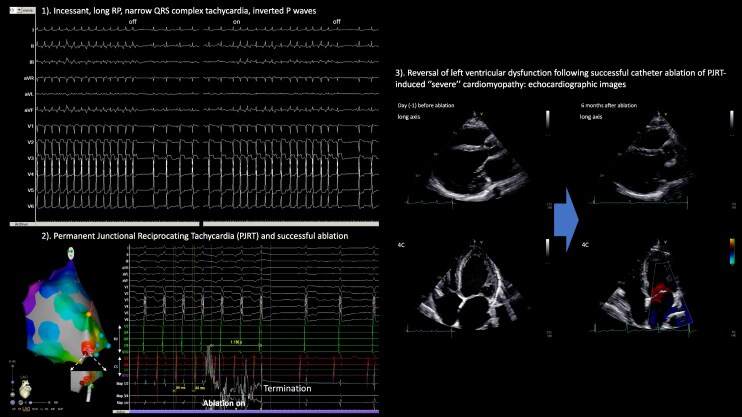


## Methods and results

### Clinical history

**Figure 1:**
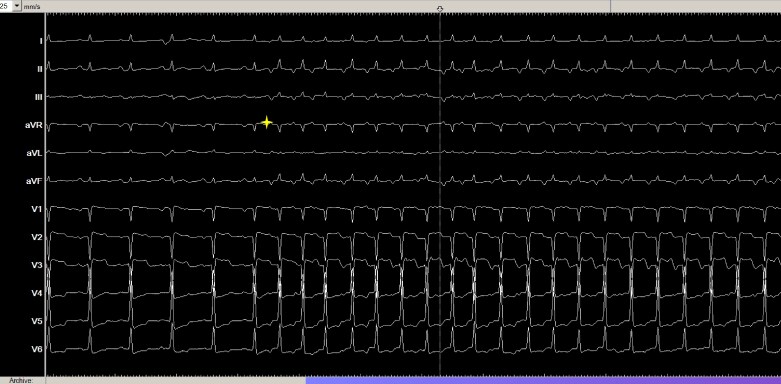
Baseline electrocardiogram showing initial sinus rhythm followed by abrupt onset of a narrow complex tachycardia. The first six beats of the electrocardiogram show a regular sinus rhythm with upright P waves in leads II, III, and aVF. This is followed by the abrupt onset (yellow star) of a narrow QRS complex tachycardia (heart rate 135/min), characterized by negative P waves in the inferior leads (II, III, aVF), suggestive of a long RP supraventricular tachycardia.

A 41-year-old patient presented with progressive exertional dyspnoea and reduced exercise capacity over several months. A 12-lead electrocardiogram (ECG) during admission showed sinus rhythm alternating with recurrent long RP narrow QRS tachycardia at a rate of 135 b.p.m. (*Figure 1*). Continuous ECG monitoring revealed the tachycardia to be almost incessant, interrupted only by brief periods of sinus rhythm (see Supplementary material online, *Figure S1*).

Transthoracic echocardiography (TTE) revealed a markedly dilated left ventricle (LV end-diastolic diameter 63 mm) with severely reduced systolic function (LVEF 14%) during tachycardia. Despite severe LV dysfunction, the patient remained haemodynamically stable without hypotension or pulmonary congestion.

Potential reversible causes, including relevant ischaemia, medication effects, electrolyte imbalance, and thyroid dysfunction, were systematically excluded. Cardiac magnetic resonance (CMR) imaging was performed before the ablation procedure to exclude structural cardiomyopathy, inflammation, or infiltrative disease.

After multidisciplinary discussion, an electrophysiological (EP) study and potential catheter ablation were recommended. Written informed consent was obtained before the procedure. The clinical timeline is summarized in *Table 1*.

**Table 1:** Timeline/care checklist

Time	Event/finding
∼6 months before admission	Young patient, gradual onset of reduced exercise tolerance and exertional dyspnoea
Admission	− Incessant long RP narrow QRS tachycardia on ECG − Severe LV dysfunction (LVEF 14%), LVEDD enlarged (63 mm)
Hospital stay	− Electrophysiologic study confirmed PJRT − Catheter ablation performed targeting posteroseptal concealed accessory pathway − Immediate termination of tachycardia − VA conduction and adenosine testing confirmed no residual pathway − (Day 2) Improvement of LVEF to 26%, LVEDD to 58 mm on post-ablation echocardiography
Discharge (Day 3)	− Asymptomatic − Further no recurrence on continuous ECG monitoring − No antiarrhythmic drug prescribed
6-month follow-up	− Patient asymptomatic, markedly improved exercise capacity − Further no arrhythmia recurrence − Echocardiography: LVEF improved to 50%, LVEDD to 52 mm

### Electrophysiological study and catheter ablation

The electrophysiological (EP) and ablation procedure was conducted by an experienced electrophysiologist (S.C., primary operator). The patient was under conscious sedation. The tachycardia was incessant and identical to the clinical tachycardia documented on surface ECG (*Figures 1*; Supplementary material online, *Figure S1*). After obtaining femoral venous access, diagnostic catheters were positioned in the high right atrium, His bundle region, right ventricular (RV) apex, and coronary sinus (CS).

**Figure 2:**
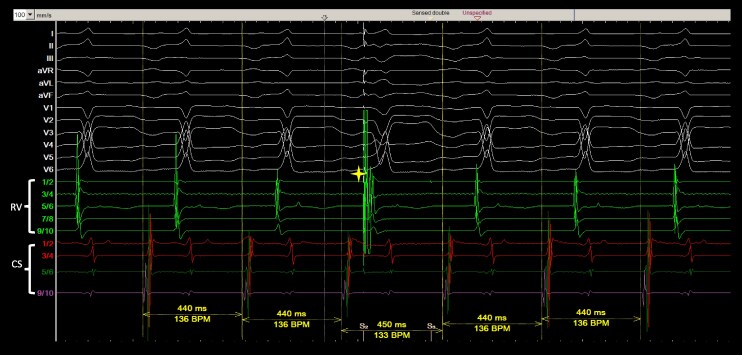
His-refractory ventricular pacing delays the subsequent atrial activation. His-refractory ventricular pacing (yellow star) delays the subsequent atrial activation, suggestive of a concealed, retrogradely conducting, decremental accessory pathway participating in the tachycardia.

During the EP study, a diagnostic catheter was indeed positioned at the His bundle region at the start of the procedure; however, it later migrated slightly towards the RV septum, which explains why distinct His electrograms are not visible in *Figure 2*. As shown in *Figure 2*, the ventricular premature beat (VPB) used for diagnostic assessment was His-refractory, as evidenced by fusion between the intrinsic QRS and the paced QRS complex, confirming that the His bundle was already depolarized at the time of stimulation. During tachycardia, the baseline A-A interval was 440 ms, and after delivery of the His-refractory VPB, the subsequent A-A interval was prolonged to 450 ms. This 10 ms A-A delay indicates that the VPB interacted with the retrograde limb of the circuit, producing decremental conduction through a slowly conducting concealed accessory pathway. These findings confirm that the observed tachycardia was mediated by a retrograde decremental accessory pathway, consistent with PJRT, and not by atypical AVNRT or atrial tachycardia.

Additional pacing was indeed performed during tachycardia. Ventricular overdrive pacing (VOP) demonstrated decremental retrograde conduction with progressive VA prolongation at faster pacing rates, consistent with a retrogradely conducting, slowly conducting accessory pathway. Because the tachycardia was nearly incessant, pacing during stable sinus rhythm could not be reliably performed, and entrainment or para-Hisian pacing were not repeated to avoid excessive tachycardia interruption or acceleration.

In this case, the His-refractory VPB response provided the most diagnostic information. The VPB produced a reproducible prolongation of the next A-A interval (440 → 450 ms), proving interaction with the retrograde limb of the circuit.

Taken together, (i) the long RP tachycardia, decremental VA conduction during VOP, (ii) His-refractory VPB response, and (iii) the point to be mentioned below, termination with ablation at the posteroseptal CS region, conclusively support the diagnosis of PJRT rather than atypical AVNRT or atrial tachycardia.

**Figure 3:**
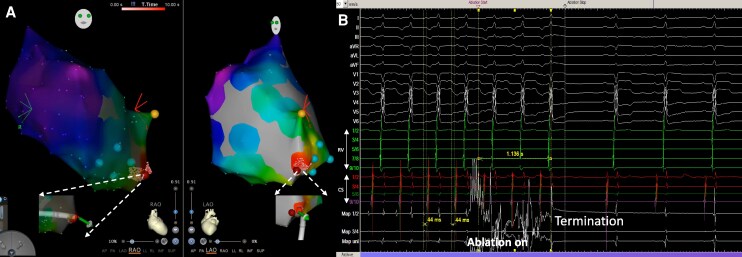
Three-dimensional electroanatomic mapping and catheter ablation of a permanent junctional reciprocating tachycardia. (*A*) During tachycardia, three-dimensional electroanatomic mapping identified the earliest atrial activation at the posteroseptal region of the right atrium near the coronary sinus ostium (red spot). White arrows indicate the position of the ablation catheter during ablation. (*B*) Radiofrequency ablation at this site resulted in immediate termination of the permanent junctional reciprocating tachycardia. Yellow point indicates the His bundle position; green points mark the coronary sinus ostium.

As shown in *Figure 3*, a three-dimensional electroanatomic map of the RA was created using the 3D-CARTO system (Biosense Webster). Activation mapping during tachycardia localized the earliest atrial activation to the posterior septal region of the RA near the coronary sinus ostium. An 8F, 3.5 mm irrigated tip ablation catheter (SmartTouch SF, Biosense Webster) was positioned at this site. Radiofrequency energy was delivered at 30 W for 60 s, with an irrigation rate of 8 mL/min and contact force maintained at 10 g. This resulted in immediate termination of the tachycardia, without any steam-pop or impedance rise.

Following ablation, a comprehensive programmed stimulation protocol was performed with and without isoproterenol infusion, which failed to induce any tachycardia. Ventricular pacing confirmed the absence of retrograde VA conduction over the accessory pathway. After a 30-min observation period, the comprehensive programmed stimulation protocol was repeated—with and without isoproterenol—and again demonstrated sustained non-inducibility and no recovery of accessory pathway conduction. Intravenous adenosine was administered only as an adjunctive confirmatory manoeuvre to assess for any residual retrograde conduction; as expected, no VA conduction was observed. The primary procedural endpoints were non-inducibility of tachycardia and absence of VA conduction, while antegrade AV nodal conduction remained intact. The procedure was completed without complications, with a total mapping time of 5 min, radiofrequency delivery time of 60 s, and minimal fluoroscopy exposure (10 s).

### In-hospital monitoring and follow-up

**Figure 4:**
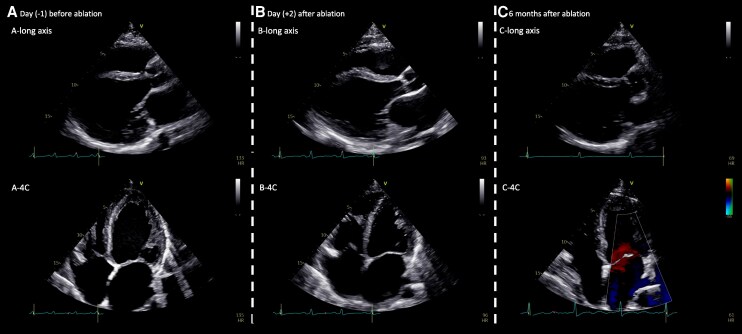
Reversal of left ventricular dysfunction following successful catheter ablation of permanent junctional reciprocating tachycardia–induced severe cardiomyopathy: echocardiographic images. (*A*) Parasternal long-axis and apical four-chamber views at presentation showing a markedly dilated left ventricle (LVEDD 63 mm) with severely reduced systolic function (LVEF 14%). (*B*) Immediate post-ablation echocardiogram demonstrating a reduction in left ventricular size (LVEDD 58 mm) and partial improvement in contractility (LVEF 26%). (*C*) Six-month follow-up echocardiogram showing near normalization of chamber dimensions (LVEDD 52 mm) and recovered systolic function (LVEF 50%).

Continuous ECG monitoring for 24 h post-ablation showed stable sinus rhythm with no recurrence. Repeat TTE on Day 2 demonstrated early improvement in LV systolic function (LVEF 26%) and reduction in LVEDD to 58 mm, indicating partial functional recovery (*Figure 4A* and *B*). The patient remained asymptomatic and was discharged on guideline-directed medical therapy for heart failure (sacubitril/valsartan 49/51 mg twice daily, bisoprolol 5 mg daily, spironolactone 25 mg daily, and dapagliflozin 10 mg daily) without antiarrhythmic medication.

At 3- and 6-month follow-up, the patient reported no recurrence of palpitation and marked improvement in exercise tolerance, with no arrhythmia recurrence on ambulatory monitoring (24-h ECG monitoring). Echocardiography showed recovery of LV systolic function (LVEF 50%) and normalization of LV dimensions (LVEDD 52 mm), consistent with reversal of tachycardia-induced severe cardiomyopathy (*Figure 4C*). Heart failure medications were subsequently tapered, and further follow-up was scheduled.

## Discussion

Permanent junctional reciprocating tachycardia is a rare form of orthodromic atrioventricular re-entrant tachycardia (AVRT), typically involving a concealed accessory pathway with slow, decremental retrograde conduction. This distinctive physiology results in a long RP interval on ECG and a tendency for incessant or frequently recurrent tachycardia, often with modest heart rates to delay clinical recognition and contribute to underdiagnosis.^4^

A key concern with PJRT is its propensity to cause TIC. Persistent tachycardia leads to progressive left ventricular systolic dysfunction and chamber dilation, mediated by adverse remodelling, impaired calcium handling, and neurohormonal activation. Importantly, TIC is often fully reversible once the arrhythmia is eliminated, underscoring the necessity of early identification and intervention.^5^ In this case, the patient presented with severely reduced LVEF (16%), highlighting the extent to which PJRT can compromise cardiac function.

While medical therapy (beta-blockers, calcium channel blockers, or class IC/III agents) may offer temporary rate control, it rarely achieves durable suppression. Catheter ablation of the accessory pathway is the treatment of choice, providing high success rates and preventing ongoing tachycardia burden.^6–8^

Our patient underwent successful radiofrequency ablation targeting the posterior septal concealed accessory pathway, with immediate termination of tachycardia and early improvement in LVEF (to 26%) on post-ablation echocardiography, suggesting rapid haemodynamic recovery.

Importantly, we performed post-ablation testing including programmed stimulation with isoproterenol, VA conduction testing, and adenosine challenge, all of which demonstrated absence of residual accessory pathway conduction. This systematic approach is essential to minimize recurrence, given that concealed decremental pathways may sometimes evade detection.

Previous studies have demonstrated that the long-term prognosis after successful ablation of PJRT is excellent, with the majority of patients achieving sustained arrhythmia freedom, recovery of ventricular function, and marked improvement in symptoms.^9–11^

At 3- and 6-month follow-up, 24-h ECG monitoring demonstrated stable sinus rhythm without recurrence of supraventricular tachycardia, and the patient remained free of antiarrhythmic medication. The patient reported complete resolution of palpitations and marked improvement in exercise capacity compared with his pre-ablation status. Echocardiography confirmed sustained recovery of LV systolic function (LVEF 50%) and normalization of chamber dimensions. This highlights the reversibility of even very severe TIC in PJRT when timely curative therapy is employed.

## Conclusion

**Figure 5:**
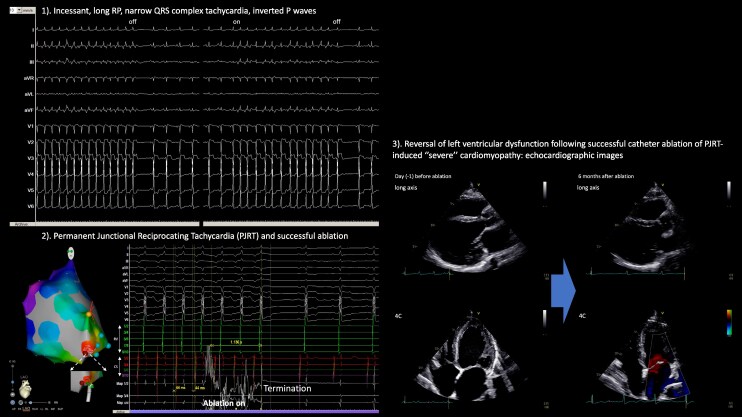
Graphic summary—severe cardiomyopathy due to permanent junctional reciprocating tachycardia: recovery after catheter ablation? (1). Incessant, long RP, narrow QRS complex tachycardia, inverted P waves. (2) Permanent junctional reciprocating tachycardia and successful ablation. (3) Reversal of left ventricular dysfunction following successful catheter ablation of PJRT-induced ‘severe’ cardiomyopathy: echocardiographic images.

The key message is illustrated in *Figure 5* (graphic summary). This case highlights the importance of recognizing PJRT as a cause of tachycardia-induced severe cardiomyopathy. Timely catheter ablation can achieve both arrhythmia cure and reversal of cardiac dysfunction, underscoring the need for early electrophysiologic evaluation in similar presentations.

## Supplementary Material

ytag089_Supplementary_Data

## Data Availability

Data are contained within the article.

## Lead author biography

Dr Shaojie Chen, MD, PhD, FESC, EHRA-Fellow (Linz/Austria 2014), EHRA fully certificated EP (Nice/France 2016), FESC (Frankfurt/Munich/Germany 2018), is currently director of Clinical Cardiac Electrophysiology at the University Medicine Greifswald, Germany. He is an EHRA-certified interventional electrophysiologist with over 10 years of experience >7000 procedures, near-zero complication rate since 2023. His practice/expertise spans cardiology, complex ablation, and modern mapping/ablation technologies and devices. He has authored 165 PubMed-listed publications (70 first/last authorship, 12/2025) in leading scientific journals. Dr Chen also serves as an Associate/Section Editor/Reviewer for major journals across cardiology/electrophysiology.
