# Supplementary material for: Severe cardiomyopathy due to permanent junctional reciprocating tachycardia: recovery after catheter ablation?—a case report
Source: Eur Heart J Case Rep. 2026 Feb 3;10(3):ytag089. doi: 10.1093/ehjcr/ytag089 (PMC13007593; doi:10.1093/ehjcr/ytag089)
Supplement: ytag089_Supplementary_Data [file ytag089_supplementary_data.docx]

Supplementary file

S-Figure 1

ECG monitoring demonstrating the incessant initiation and spontaneous termination of the clinical tachycardia


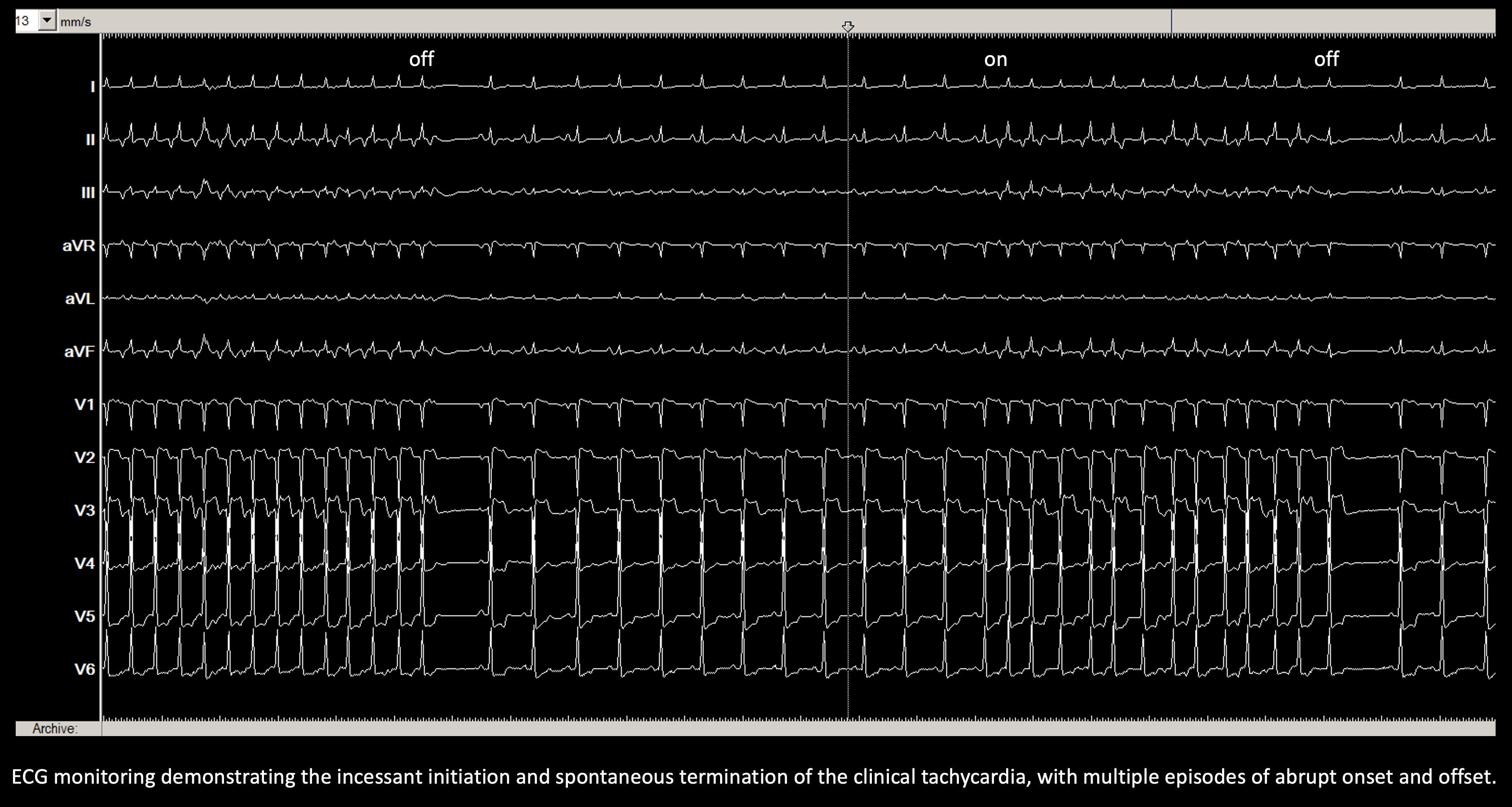


ECG monitoring demonstrating the incessant initiation and spontaneous termination of the clinical tachycardia, with multiple episodes of abrupt onset and offset.
